# Ill-Shaped Noses, and Other Irregularities of the Human Body Not Morbid

**Published:** 1884-12

**Authors:** 


### ILL-SHAPED NOSES AND OTHER IRREGULARITIES OF THE HUMAN BODY NOT MORBID.

*Editors Atlanta Medical and Surgical Journal:*

As we walk the streets we meet many noses; some good comely noses, and some, though uncomely, subserve all of the purposes of their owner. Among this multitude of noses that look well enough to the passer-by, very few, upon close inspection, are set centrally on one's visage, or are of orthodox form, size and proportions. It might be a little hazardous, however philosophic our intentions, to investigate the nose of Tom, Dick, or Harry, as we meet those worthies on the street; but within the metes and bounds of safe investigation it will be found that nine noses out of ten (my statistics are not exact) of said organs lean too much to the left, or the right, are too big or too little, too long or too short, too high or too low, too flat or too sharp, too thin or too thick; not to emphasize (for the dangers involved) their being too red, or too blue sometimes. Nevertheless, under all of these disabilities, the little organ serves the purpose of its owner with due fidelity in smelling, breathing, blowing and sneezing as fully as if it had been chiseled by an artistic sculptor.

There are many other irregularities in the human body which, though equally abnormal, are not morbid. To say nothing about hands, feet, eyes, ears, or mouth; Madames Bouvain and LaChapelle and others in their line, have given, or could give numberless cases of the irregular development of the external and internal genitalia of their patients, without harmful results, or the necessity of gynecological intervention, as evidenced by conception, gestation and parturition, taking their normal course with best results. There are irregularities of position and development and disease of certain organs that require the interposition of art, and it is pleasant to know that we have surgeons of our own, who are an honor to their calling and a blessing to the country, and who compare well with the best gynecologists of other lands. But it is not to be concealed that some well-meaning medical men seem forgetful of the resources of nature, and that no harm is intended by that divinity when she sports a little with a nose or a uterus, and that the uterus leaning a little to the right or left, front or rear, don't always require the interposition of art any more than a bent nose does. Is it not best to stand back and not familiarize ourselves too much with other people's noses or uteri; for the one might be dangerous, the other should be sacred. *Verbum sat.* * * * * * * *

But please allow a little more about intermeddling, as it is not drawn from imagination; Mrs.——, young and healthy had (her Doctor said) uterine disease calling for surgical interference. Before such measures were taken, an appeal was made privately to an old conservative medical friend, who advised delay and gentle remedies. In due time she was happily delivered of a healthy child, which in less than two years had a successor. Another, Mrs.—— could not menstruate, as previously, and was in trouble about it. A doctor with strong gynecological tendencies and aspirations found the os uteri closed up and art called for. The husband says the doctor used "something like a glove stretcher." A few hours brought forth an abortion, to the dismay of the doctor and distress of the patient. Miss A. has incipient tuberculosis and Miss B. has mental peculiarities; both of them just verging into womanhood. Manipulate their genitals is the prescription!! Is there not a moral as well as physical evil, in thus outraging modesty by unnecessary and familiar interference with the female organs of generation? Few it is believed err so grossly. But does not fashion drift towards undue interference in this direction?

November, 1884. A CONVERT, M. D.

